# Complete Genome Sequences of Caldicellulosiruptor acetigenus DSM 7040, Caldicellulosiruptor morganii DSM 8990 (RT8.B8), and Caldicellulosiruptor naganoensis DSM 8991 (NA10)

**DOI:** 10.1128/mra.01292-22

**Published:** 2023-02-01

**Authors:** Ryan G. Bing, Daniel J. Willard, Mohamad J. H. Manesh, Tunyaboon Laemthong, James R. Crosby, Michael W. W. Adams, Robert M. Kelly

**Affiliations:** a Department of Chemical and Biomolecular Engineering, North Carolina State University, Raleigh, North Carolina, USA; b Department of Biochemistry and Molecular Biology, University of Georgia, Athens, Georgia, USA; Portland State University

## Abstract

The genome sequences of three extremely thermophilic, lignocellulolytic *Caldicellulosiruptor* species were closed, improving previously reported multiple-contig assemblies. All 14 classified *Caldicellulosiruptor* spp. now have closed genomes. Genome closure will enhance bioinformatic analysis of the species, including identification of carbohydrate-active enzymes (CAZymes) and comparison against other *Caldicellulosiruptor* species and lignocellulolytic microorganisms.

## ANNOUNCEMENT

Extremely thermophilic *Caldicellulosiruptor* bacteria have the ability to degrade and metabolize complex polysaccharides from plant biomass, including cellulose, hemicellulose, pectin, and starch (1–5). There are 14 classified *Caldicellulosiruptor* strains, all with genome assemblies, but three remained unclosed: Caldicellulosiruptor acetigenus DSM 7040 (6), Caldicellulosiruptor morganii DSM 8990 (7), and Caldicellulosiruptor naganoensis DSM 8991 (7). Recently, *C. kristjanssonii* and *C. lactoaceticus* were reclassified as strains of *C. acetigenus*, despite the DSM 7040 assembly comprising 37 contigs (8). Here, the remaining classified *Caldicellulosiruptor* genomes were closed to improve comparisons both within and outside the genus. Cultures originally acquired from the DSMZ (German Collection of Microorganisms and Cell Cultures) were maintained in modified defined DSM 516 medium with 15% glycerol at −80°C (2). Cultures were anaerobically grown at 75°C in modified DSM 516 medium.

All methods were carried out per the manufacturer’s instructions unless otherwise noted. The NEB Monarch genomic DNA purification kit (New England Biolabs, USA) was used to purify genomic DNA from liquid cultures using the tissue lysis buffer and lysozyme. DNA was barcoded and sequenced using the Oxford Nanopore Technologies (UK) ligation sequencing kit (SQK-LSK109), the native barcoding expansion kit 1-12 (EXP-NBD104), and a R9.4.1 flow cell (FLO-MIN106D) on a MinION Mk1B device; no DNA size selection was used. A Qubit 4.0 fluorimeter with the 1× double-stranded DNA (dsDNA) high-sensitivity (HS) assay kit (Q33231; Invitrogen) was used to quantify the DNA. The barcoded libraries were pooled and sequenced for 72 h. MinKNOW v5.1.8 (Oxford Nanopore Technologies) was used with Guppy v6.1.5 live high-accuracy GPU base calling. MinIONQC was used to evaluate the read quality (9). The long-read metrics are provided in Table 1.

**TABLE 1:** Previous and new assembly information for three *Caldicellulosiruptor* species

Data type	Characteristic	Data for strain:		
		Caldicellulosiruptor acetigenus DSM 7040	*Caldicellulosiruptor morganii* DSM 8990	*Caldicellulosiruptor naganoensis* DSM 8991
Best previous assembly (multiple contigs)	Assembly size (bp) No. of contigs Contig *N*_50_ (bp) GenBank assembly accession no.	2,589,957 37 142,649 GCA_000421725.1	2,488,483 2 2,097,568 GCA_000955745.1	2,514,985 12 1,295,458 GCA_000955735.1
				
New assembly information (completely closed)	Genome size (bp) GC content (%) Coverage (×) Long reads All reads Reads mapped (%) Completeness (%) Contamination (%) No. of predicted genes BioProject accession no. BioSample accession no. GenBank assembly accession no.	2,739,610 36.33 237 1,228 99.83 99.52 0.00 2,664 PRJNA904341 SAMN31840423 CP113866	2,474,467 36.50 123 1,776 99.94 95.07 1.11 2,413 PRJNA904341 SAMN31840452 CP113865	2,494,610 35.30 253 1,783 99.65 96.01 2.39 2,477 PRJNA904341 SAMN31840465 CP113864
				
Reads and read metrics Nanopore long reads	SRA accession no. No. of reads *N*_50_ (bp) Total reads (bp)	SRX18439046 316,279 15,829 762,266,968	SRX18439047 94,245 6,161 351,989,952	SRX18439048 76,424 22,843 667,303,418
				
Short reads (data published previously)	SRA accession no. JGI Code Project no. GOLD accession no. Reference	SRX301888 CalaceDSM7040_FD 1013583 Gp0013000 6	SRX1880054 CalspRB8DSM8990_FD 1017510 Gp0035174 7	SRX1879649 ThecelNA1DSM8991_FD 1017504 Gp0035078 7

The long reads were trimmed during base calling using Guppy and filtered using NanoFilt v2.8.0, with read cutoffs of 500 bp (length) and Q10 (quality) (10). The Flye assembler (v2.9.1 for *C. acetigenus*; v2.8.1 for *C. morganii*/*C. naganoensis*) was used to assemble and circularize the genomes from the ONT long reads (11). The Circlator v1.5.5 (12) FIXSTART task was used to rotate the circularized assemblies to start at the *dnaA* gene, which was predicted using Prodigal v0.5.2 (13). The assemblies were error corrected using both the Nanopore long reads and previously published Illumina short reads (6, 7) (see Table 1) using Pilon v1.24 (14). The short reads were trimmed using Trimmomatic (v0.36 for *C. acetigenus*; v0.39 for *C. morganii*/*C. naganoensis*) with the following parameters: TruSeq3-PE.fa adapter sequence, 2 seed mismatches, 30 palindrome clip threshold, 10 simple clip threshold, leading and trailing set to 3 bp, sliding window set to 4 bp and Phred 15, minimum length set to 36 bp, and average quality set to Phred 30 (15). Read mapping was done with Bowtie2 (v2.4.5 for *C. acetigenus*; v2.2.5 for *C. morganii*/*C. naganoensis*) (16) for the short reads and GraphMap v0.5.2 (17) for the long reads; SAMtools (v1.15.1 for *C. acetigenus*; v1.13 for *C. morganii*/*C. naganoensis*) (18) was used to sort and index the sequences. Lastly, the assemblies were polished using BWA v0.7.17 (19), and the short reads were mapped using Polypolish v0.5.0 (20). The finalized assemblies were evaluated using Pilon (with –fix flag of “none”) (14), QUAST v5.2.0 (21), and CheckM v1.2.1 (with a reduced genome tree) (22) (Table 1). No major misassembles were detected using Pilon. PGAP v2022-08-11.build6275 (23) was used to annotate the final genomes. Unless indicated otherwise, default parameters were used for all software.

### Data availability.

The GenBank, BioProject, and BioSample accession numbers are provided in Table 1 for the Nanopore long reads and new genome assemblies. Also provided in Table 1 are the accession numbers for the previously published short reads used and the best previous assembly for the microorganism. Note that the raw short reads were downloaded from the JGI GOLD portal and not NCBI, but both links are provided.
